# The Communication between Ocular Surface and Nasal Epithelia in 3D Cell Culture Technology for Translational Research: A Narrative Review

**DOI:** 10.3390/ijms222312994

**Published:** 2021-11-30

**Authors:** Malik Aydin, Jana Dietrich, Joana Witt, Maximiliane S. C. Finkbeiner, Jonas J.-H. Park, Stefan Wirth, Christine E. Engeland, Friedrich Paulsen, Anja Ehrhardt

**Affiliations:** 1 Laboratory of Experimental Pediatric Pneumology and Allergology, Center for Biomedical Education and Research, School of Life Sciences (ZBAF), Department of Human Medicine, Faculty of Health, Witten/Herdecke University, 58448 Witten, Germany; malik.aydin@uni-wh.de; 2 Center for Child and Adolescent Medicine, Center for Clinical and Translational Research (CCTR), Helios University Hospital Wuppertal, 42283 Wuppertal, Germany; stefan.wirth@uni-wh.de; 3 Institute of Functional and Clinical Anatomy, Friedrich Alexander University Erlangen-Nürnberg, 91054 Erlangen, Germany; jana1.dietrich@fau.de; 4 Department of Ophthalmology, University Hospital Düsseldorf, Heinrich-Heine-University, 40225 Düsseldorf, Germany; joana.witt@med.uni-duesseldorf.de; 5 Virology and Microbiology, Center for Biomedical Education and Research (ZBAF), Department of Human Medicine, Faculty of Health, Witten/Herdecke University, 58453 Witten, Germany; maximiliane.finkbeiner@uni-wh.de (M.S.C.F.); christine.engeland@uni-wh.de (C.E.E.); 6 Department of Otorhinolaryngology, Kath. Krankenhaus Hagen gGmbH, St.-Josefs-Hospital, Faculty of Health, Department of Human Medicine, Witten/Herdecke University, 58097 Hagen, Germany; jonas.park@uni-wh.de; 7 Department of Topographic Anatomy and Operative Surgery, Sechenov University, 119146 Moscow, Russia

**Keywords:** ocular surface epithelium, goblet cells, nasal epithelium, 3D cell culture, infection

## Abstract

There is a lack of knowledge regarding the connection between the ocular and nasal epithelia. This narrative review focuses on conjunctival, corneal, ultrastructural corneal stroma, and nasal epithelia as well as an introduction into their interconnections. We describe in detail the morphology and physiology of the ocular surface, the nasolacrimal ducts, and the nasal cavity. This knowledge provides a basis for functional studies and the development of relevant cell culture models that can be used to investigate the pathogenesis of diseases related to these complex structures. Moreover, we also provide a state-of-the-art overview regarding the development of 3D culture models, which allow for addressing research questions in models resembling the in vivo situation. In particular, we give an overview of the current developments of corneal 3D and organoid models, as well as 3D cell culture models of epithelia with goblet cells (conjunctiva and nasal cavity). The benefits and shortcomings of these cell culture models are discussed. As examples for pathogens related to ocular and nasal epithelia, we discuss infections caused by adenovirus and measles virus. In addition to pathogens, also external triggers such as allergens can cause rhinoconjunctivitis. These diseases exemplify the interconnections between the ocular surface and nasal epithelia in a molecular and clinical context. With a final translational section on optical coherence tomography (OCT), we provide an overview about the applicability of this technique in basic research and clinical ophthalmology. The techniques presented herein will be instrumental in further elucidating the functional interrelations and crosstalk between ocular and nasal epithelia.

## 1. Historical Introduction into the Ocular and Nasal Communication

Can one cry on command? Can one shed tears without crying? Are there false tears? From a purely physiological point of view, the answer is simple: when someone cries, saline tear fluid flows down the cheek [1,2]. Most of this is formed by the lacrimal glands located in the temporal upper quadrant on and at the eye bulb and passes as ‘used tear fluid’ through the draining tear ducts, which consist of the lacrimal puncta, lacrimal tubules, lacrimal sac, and nasolacrimal duct, into the nose or the lower nasal duct [3,4]. Thus, colloquially, ‘Rotz (=snot) & Wasser (=water) heulen (=cry)’ is commonly used as a traditional idiom in German culture, which also corresponds to crying the eyes out [1].

Thus, even this simple idiom implies a connection between the eye and nose.

Until well into the 16th century, it was assumed that tears were a product of the brain [3]. It was not until 1574 that the Italian Giovanni Battista Carcano Leone described the draining tear ducts quite precisely [5]. Together with the investigations of the Dane Niels Stensen (1662), after whom the main excretory duct of the parotid gland is named (Stenon’s duct, Ductus Stenonius), on tear secretion, a plausible understanding of the entire lacrimal system emerged [6]. In 1755, this was completed by the German anatomist and botanist Johann Gottfried Zinn after which the ZINN tendon ring (annulus tendineus communis), the common origin of all the external eye muscles except the obliquus inferior muscle, is named [7,8].

Nevertheless, the mechanisms that ensure tear transport through the draining lacrimal ducts—and thereby link eye and nose—are still not fully understood. Although there are currently elaborated morphological studies, unfortunately, there is still a significant lack of functional insights. The numerous existing hypotheses reflect the complex structure of the nasolacrimal ducts and show that tear transport is a complex process [4,9].

Considering these complex mechanisms and the close communication between the eyes and the nose, with this review, we aim at presenting distinct cell culture methods, which were previously published, and highlighting their applicability in translational contexts. The interconnection between the ocular and nasal epithelia is important to study the etiopathogenesis of diseases affecting these tissues and to develop therapeutic algorithms. A summary on current cell culture models of these epithelia and important technical notes and nuances will guide experimentalists to work with these models in their laboratories. Moreover, an introduction to current 3D cell culture techniques for conjunctival, corneal, nasolacrimal, and nasal epithelial cells are an important advancement discussed herein. In addition to the morphological preservation of these difficult-to-cultivate but valuable cells in a physiological context, some disease pathologies affecting these tissues will be illustrated. Exemplarily, adenoviruses (AdV) and measles virus (MeV) as important pathogens but also allergens during the pathogenesis of rhinoconjunctivitis are discussed. At the end, we will provide an experimental outlook, which may serve as a scaffold for scientists aiming to work with these cell culture models to study physiological and pathological aspects of ocular and nasal epithelia. 

## 2. Morphology and Physiology of the Ocular Surface Epithelium, Nasolacrimal Ducts, and Nasal Epithelium

**Figure 1:**
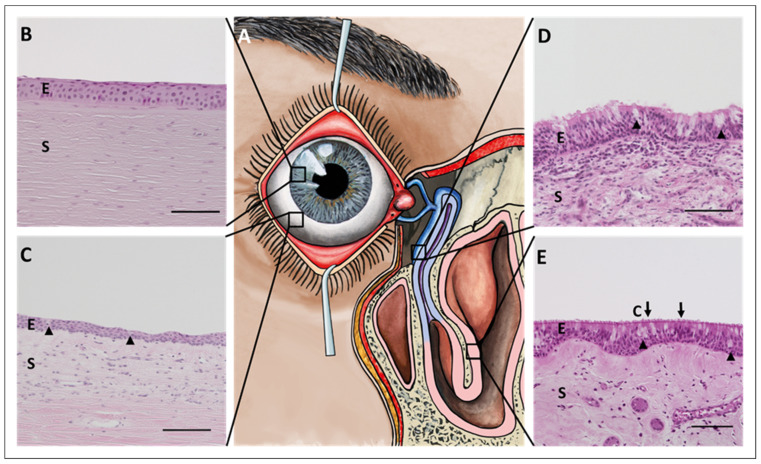
Morphology and physiology of the ocular surface epithelium, nasolacrimal duct, and nasal epithelium. (**A**) Schematic drawing of the anatomical connection between the ocular surface and the nasal cavity via the nasolacrimal duct. Illustration by Jörg Pekarsky, Institute of Functional and Clinical Anatomy, FAU Erlangen-Nürnberg, (**B**) Hematoxylin and Eosin staining (HE) of the human cornea, (**C**) the human bulbar conjunctiva, (**D**) the human nasolacrimal duct, and (**E**) the human nasal cavity. E: epithelium, S: stroma; arrowheads mark goblet cells, C: cinocilia marked by arrows; Magnification is 200× with 100 µm scale bar.

This section summarizes the native anatomical morphology as well as the physiological function of the ocular surface epithelium (cornea and conjunctiva), the efferent tear ducts (nasolacrimal ducts), and the nasal cavity (Figure 1A). These anatomical structures are in direct physical connection with each other and represent the physiological tear out-flow from the formation of the tear film at the ocular surface via the drainage through the nasolacrimal ducts to the end point of the ’used tear fluid´ in the nasal cavity [3,4]. Tears can be considered a carrier of biological information exchanged between the eye and the nose, a tool of communication. The tear film covers the ocular surface epithelium. It provides nutrients, hydration, and oxygen, smoothens the epithelial surface, forms an essential part of the refractive optical system, and protects against pathogens (e.g., viruses) [10]. Of note, the retina and vitreous body are not directly addressed in this review. However, the retina represents a particular layer of the eye and their corresponding retinal pathologies including retinitis pigmentosa punctata albescens (RPA) which may lead to vision loss and blindness (reviewed in [11,12]) [13]. Exemplarily, the work of Donato and colleagues examines variants in four known genes that trigger RPA and provides important clues to the etiopathogenesis of the disease [14]. 

### 2.1. Cornea

The cornea is a specialized optical tissue with transparent properties that enables light transmission into the inner eye. The cornea covers the central area of the ocular surface (our window to the outside world) and extends to the limbus, the transition zone between the cornea and the conjunctiva. With regard to the communication between the ocular surface and the nose, the cornea lies at the beginning of the communication. 

Current understanding indicates that the cornea includes five different layers [15]:

- Corneal epithelium
- Bowman´s membrane
- Stroma (with Dua’s layer)
- Descemet´s membrane
- Corneal endothelium

The corneal epithelium itself consists of five to six layers of non-keratinized squamous epithelial cells that can be further divided into basal, wing, and superficial cells (from posterior to anterior) (Figure 1B) [16]. 

The superficial cells are lined by microplicae at the tips of which the glycocalyx is anchored, mainly in the form of the membrane-anchored mucins MUC1, MUC4, and MUC16 (see below), which are part of the mucosal component of the tear film. The epithelial cells are connected through tight junctions, building an effective barrier against pathogens, environmental debris, and fluids [10]. Progenitor cells, also defined as limbal stem cells, are located in the limbal area and continuously replace cells lost through physiological turnover by differentiation and maturation processes [16,17]. The basal cells of the corneal epithelium rest on a basement membrane, which is attached to the Bowman’s membrane posteriorly. The corneal stroma consists of highly regular and orthogonally arranged lamellae composed of type I, type V, and type VI collagen fibers [18]. In addition, keratocytes are sparsely distributed within this collagenous matrix [19]. Located posterior to the stroma is the Descemet membrane, which serves as the basement membrane for the corneal endothelium. The endothelium consists of a single layer of hexagonal cells that prevent passive diffusion of the aqueous humor into the stroma and thus stromal swelling [20].

This basement membrane encompasses the extracellular matrix secreted by the corneal endothelial cells. Furthermore, the posterior corneal endothelium consists of a single layer of hexagonal cells that prevent passive diffusion of the aqueous humor into the stroma and thus stromal swelling [20]. Due to their limited regenerative capacity, endothelial cell loss needs to be compensated by the expansion (polymegathism) of neighboring cells [20,21]. Of note, when the enlarged endothelium is no longer able to maintain fluid balance, the resulting stromal swelling leads to loss of transparency, corneal edema, and visual impairment [20].

### 2.2. Conjunctiva

The conjunctiva is a transparent mucous membrane that covers the main part of the ocular surface. It consists of an epithelium with secretory goblet cells, covered by the tear film, and an underlying stroma (Figure 1C). Similar to the cornea, the conjunctiva is at the beginning of the communication between the ocular surface and the nose. Since the conjunctiva actively forms parts of the tear film, especially the mucin component, messenger substances could be secreted here, which then have an effect on the nasal epithelia.

The conjunctival epithelium in general is formed by two main cell types: the conjunctival epithelial cells and the conjunctival goblet cells. The epithelium is a multilayered unkeratinized squamous epithelium with microplicae (just as in the cornea, the microplicae are covered with a felt of membrane-bound mucins, MUC1, MUC4, and MUC16 [22]) and can be subdivided into basal, intermediate, and superficial cells. The conjunctival goblet cells produce secretory mucins, part of the mucin component of the tear film lubricating the ocular surface and thus maintaining the integrity of the entire eye [23,24,25]. The stroma of the conjunctiva consists of loose connective tissue. It contains defense cells, mast cells, and abundant blood vessels, which form a marginal loop network at the limbus corneae. In about one third of adult people, it harbors the conjunctiva-associated lymphoid tissue (CALT) that plays an important role in the immune control of the eye by activating the immune system in case of pathogen infiltration by reacting mainly with the differentiation of immunoglobulin (Ig)A producing plasma cells [26,27].

The conjunctiva begins at the corneoscleral limbus, extends to the fornix of the eye, and covers the inner layer of the eyelid to the lid margin. As each of these different regions have specific characteristics, the conjunctiva can be divided into three areas:

- Bulbar conjunctiva
- Fornical conjunctiva
- Palpebral conjunctiva

In detail, each area is exposed to different stress factors and has a distinct primary function. Thus, in each area, the non-keratinized epithelium exhibits a slightly different structure in terms of epithelial cell shape and stratification as well as goblet cell density [28]. The bulbar conjunctiva covers the sclera of the eyeball and is loosely attached to it. Here, the conjunctival epithelium consists of a stratified squamous epithelium with six to nine layers [29,30]. The fornical conjunctiva represents the transition between the bulbar and palpebral conjunctiva. In this area, the conjunctiva is highly elastic and forms folds that ensure the flexible movement of the eyeball and represents a reservoir for the tear film, as the excretory ducts of the lacrimal glands are located there [31]. In addition, the cylindrical epithelium reaches two to three layers. Moreover, the palpebral conjunctiva can be subdivided into the marginal, tarsal, and orbital conjunctiva, with the tarsal conjunctiva tightly attached to the tarsal plate and the marginal conjunctiva close to the lid margin. The tarsal conjunctiva consists of two to three layers of cuboidal epithelium, whereas the marginal conjunctiva is a stratified squamous epithelium [29,30]. 

Goblet cells are found in all areas of the conjunctiva, but with unequal distribution, with a higher density in the inferior than the superior conjunctiva as well as at the nasal side [28]. In areas of low density, goblet cells occur as solitary cells, while in areas of higher density, additional clusters are detected. Functionally, goblet cells produce and secrete gel-forming mucins (especially MUC5AC), but also trefoil factor family (TFF) peptides such as TFF3 [24,32]. Both are essential constituents of the aqueous component of the tear film. The conjunctival goblet cells as well as the conjunctival epithelium are derived from the same bi-potent progenitor cells [33]. However, it is still under discussion whether these progenitor cells are distributed uniformly or locally throughout the conjunctiva [33,34,35].

### 2.3. Draining Tear Ducts

The efferent ducts build the direct physical connection between the eye and the nose. They form the lacrimal drainage system and are responsible for the transportation of the tears from the ocular surface to the nasal cavity [4,36]. The ‘used tear fluid’ is collected at the nasal side of the eye and is drained into the inferior nasal meatus of the nose through the lacrimal puncta (upper and lower), the lacrimal canaliculi (upper and lower), the lacrimal sac, and the nasolacrimal duct [3,4]. The lacrimal puncta, as well as the lacrimal canaliculi, consist of a multilayered non-keratinized squamous epithelium. In contrast, the lacrimal sac and the nasolacrimal duct are lined by a double-layered non-keratinized epithelium that rests on a broad basement membrane (Figure 1D) [36].

The superficial epithelial layer of the lacrimal sac and the nasolacrimal duct consists of columnar epithelial cells with microvilli that are attached to the basal-cell layer. Within the epithelium of the lacrimal sac and the nasolacrimal duct, goblet cells are integrated as solitary cells or as intraepithelial mucous glands [36]. These goblet cells have been shown to produce and secrete mucins, particularly MUC5B and MUC2 but also MUC5AC, as well as TFF peptides, e.g., TFF1 and TFF3 [32,37].

Particularly in the lower section of the nasolacrimal duct, individual kinocilia-bearing epithelial cells are also present in the epithelium [36]. Absorption experiments in rabbits show that components of the transported tear fluid can be reabsorbed in the nasolacrimal duct [38], (reviewed in [39]), which allows the hypothesis that reabsorption may represent a feedback signal for tear production. Mucosa-associated-lymphoid tissue (MALT) is referred to as TALT = tear duct-associated lymphoid tissue in the draining tear ducts, and is also present in approximately one-third of adult subjects [40,41], (reviewed in [39]). After its description, it was combined with the CALT under the name EALT = eye associated-lymphoid tissue [26]. In addition to free intra- and subepithelially localized defense cells, the epithelium of the lacrimal sac and nasolacrimal duct also produces a whole arsenal of antimicrobial peptides that serve the immune defense in the ‘closed’ (located in a bony canal) system of the draining lacrimal ducts [42]. The epithelium of the lacrimal sac and nasolacrimal duct lies on a vascular system of specialized vessels that is comparable to an erectile tissue [43] and is functionally involved in tear transport [44].

### 2.4. Nasal Cavity

The nasal cavity represents the internal part of the nose and is separated into two almost symmetrical halves by the nasal septum (Figure 1E). The nasal cavity is the recipient of biological information through the tears that enter the inferior nasal meatus from the nasolacrimal duct. Biological material that has entered the tear film at the ocular surface is now released into the nasal cavity. Besides the entry of biological material with the tears, the nasal cavity is also the entry site for inhaled air and serves the sense of smell, filtering, warming, and humidifying the inhaled air while absorbing water, gases, and particles. It acts as a physical barrier against debris and pathogens [45]. Structurally, the nasal cavity can be divided into three different areas:

- Cutaneous region
- Olfactory region
- Respiratory region

In detail, the nasal inlet is covered by the keratinized, multilayered squamous epithelium (epidermis). Here, sweat and sebaceous glands, as well as vibrissae hairs, are located. The olfactory region is a smaller area in the superior nasal cavity and is lined with the olfactory epithelium [46]. The main area is built by the respiratory epithelium composed of a typical epithelium and a transitional epithelium. Both types consist of basal and superficial cells. The anterior third of the nasal cavity is lined by the transitional epithelium that is characterized by stratified, non-ciliated, cuboidal to low columnar cells with microvilli [45]. In contrast, the typical respiratory epithelium consists of stratified, ciliated columnar epithelial cells [45]. In this area, the synchronized movement of the cilia (kinocilia) serves to transport the covering mucus and all bound particles and pathogens towards the pharynx. Similar to the nasolacrimal ducts, the respiratory epithelium rests on a broad basement membrane [46]. Below the epithelium and attached to its basement membrane is the nasal stroma, which contains fibroblasts, immune cells such as lymphocytes and mast cells as well as a dense specialized vasculature in the form of a cavernous body, nerves, and submucosal (nasal) glands [45]. The nasal glands together with intraepithelial goblet cells produce the seromucous secretion of the nasal mucosa that moisten the nasal epithelium, bind and transport foreign particles. These goblet cells have been shown to produce and secrete mucins, particularly MUC5AC and MUC2, as well as TFF peptides, e.g., TFF 1 and TFF3 [47,48,49,50]. The goblet cells are distributed throughout the respiratory epithelium with a higher density in the posterior than in the anterior part [51].

## 3. Development of 3D Cell Culture Models

Vision loss but also chronic nasal symptoms affect a person’s quality of life [52,53]. Therefore, it is important to advance research in this area to provide better treatment options and alternatives to such patients. With regard to this, 3D cell culture models provide a basis for better understanding of the pathogenesis of diseases and may result in the development of therapies for such patients [54]. In addition, cell-cell or cell-matrix interactions can be studied [54,55]. Due to the 3D cell culture methodology, the different cell types have good access to nutrients and oxygen in this in vitro environment [54,56]. Organotypic cell culture models arrived in cell culture banks several years ago. Their advantage is that cells are extracted from tissues or from biopsies and seeded on semipermeable membranes after few passages [57,58]. In the appropriate cell culture media with growth factors and other supplements, the cells differentiate and acquire some organotypic phenotype, wherein cell-cell or cell-matrix interactions can be studied, as well as pathogenesis of e.g., viral infection [54,57,58,59,60,61,62,63]. Therefore, the following sections first describe the current 3D models for each compartment individually. For investigating the connection and communication between the ocular surface and the nasal epithelia, either (i) complex 3D models combining the current individual models could be used, or (ii) the same experimental question could be investigated simultaneously on the individual 3D models and the results then correlated.

### 3.1. 3D Cell Culture of Corneal Epithelium

The cornea is transparent and non-vascularized [54]. It is anatomically composed of five distinct layers [15,54] with different cell types (Section 2.1) (reviewed in [54]). A 3D in vitro model of the cornea would therefore need to account for these different cell types and layers, which is not easy to implement [54]. The cornea functions as a refracting lens, which focuses the visible light and acts as a protector of the inner part of the eye (reviewed in [64,65]). Morphologically, between the anterior corneal epithelial and the posterior corneal endothelial parts, the corneal stroma constitutes the main layer (reviewed in [64]). To date, there is increasing awareness of the complexity of the corneal stroma and hierarchical organization of the collagen fibrils in the stroma. Previously, these were not or insufficiently well studied, and it is important to explore this ultrastructural part of the eye in more detail (reviewed in [64,65]) [66,67,68,69,70]. As described below in more detail, optical coherence tomography (OCT) is an important technique in visualizing also the ultrastructure of the cornea [71,72]. Exemplarily, Napoli and colleagues have used the OCT to explore the corneal stromal striae in *Ovis aries* [73]. 

Epithelial stroma cultures, epithelial stroma cultures with nerves or corneal endothelial stroma cultures were already described by different authors (reviewed in [54]). In the first culture type, cell-cell interactions between corneal fibroblasts and epithelial cells are analyzed, which can also be used as a so-called wound and healing model of the cornea [54]. 3D cell culture models with a single cell type can be established for example within an air-liquid interface model, and drug effects, injury, or chemical noxious agents can be studied [74,75,76,77], (reviewed in [54]). Pluripotent stem cells can differentiate into any cell type and acquire so-called organ-like structures [78]. Organoid models are certainly an important milestone in experimental research, as these so-called mini-organs incorporate an in vivo-like morphology and physiology. Organoids are defined as a cell cluster of organ-specific cell types originating from stem or progenitor cells that self-organize and group to resemble an organ or organ-like structures [78]. Due to their organ-like structures in culture dishes, hypotheses related to organ physiology and dysfunction can be studied in vitro [78]. Moreover, drug testing can be also performed in such models at a scale that cannot normally be applied in human trials [78]. Foster and colleagues were finally able to culture corneal organoids from human induced-pluripotent stem cells (iPSCs) containing the known three different cell types with the corresponding epithelial, stromal, and endothelial cell marker expression [54,79]. In detail, first, iPSCs were induced/stimulated, which consequently led to neural induction and finally to induction at day 12 and maturation at day 20. This was followed by corneal selection at day 30 and eventual maturation at >120 days [79]. In addition, so-called organ chips may also represent an interesting model, which can be alternatively used in the laboratory to study eye disorders in vitro [52,80]. Rötzer et al. (2019) recently presented an interesting ex vivo slice culture model using ‘thicker’ (300 µm) tissue sections of donor eyelids in culture. They were able to study the regulation of cell cohesion in meibocytes on the slice cultures for up to 6 days [81]. A similar model could be envisioned for the cornea. For all in vivo cultivations, one should be aware of how important mechanical influences on culture conditions are as well. Hampel et al. (2018) showed that shear stress as it occurs at the corneal epithelium during blinking exerts marked effects on corneal epithelial cells, such as changes in cellular morphology and the expression of cell junctions [82].

**Figure 2:**
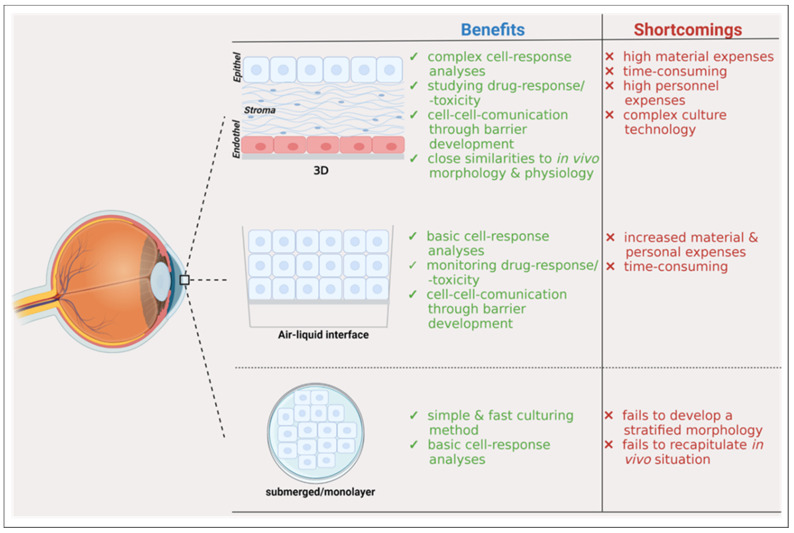
Summary of different corneal cell culture models with their benefits and shortcomings. This figure was adapted from [83], and was created with BioRender.com.

Figure 2 summarizes the benefits and shortcomings of distinct corneal cell culture models.

### 3.2. 3D Cell Culture of Epithelia with Goblet Cells

The epithelium of the conjunctiva, lacrimal sac, nasolacrimal duct, and nasal epithelia contain specialized mucus-secreting goblet cells. Moreover, nasal mucosa grafts were used for conjunctiva reconstruction after extensive damage with satisfactory results [84,85]. Depending on the site of tissue harvest in the nasal cavity, as well as in the conjunctiva, the amount of goblet cells can be ‘chosen’ [28,51]. Therefore, cell culture conditions may be similar, in particular with regard to isolation, differentiation, and/or maintenance of goblet cells. In this section, challenges, and achievements to establish 3D in vitro cultivation of epithelia with goblet cells are compiled.

**Figure 3:**
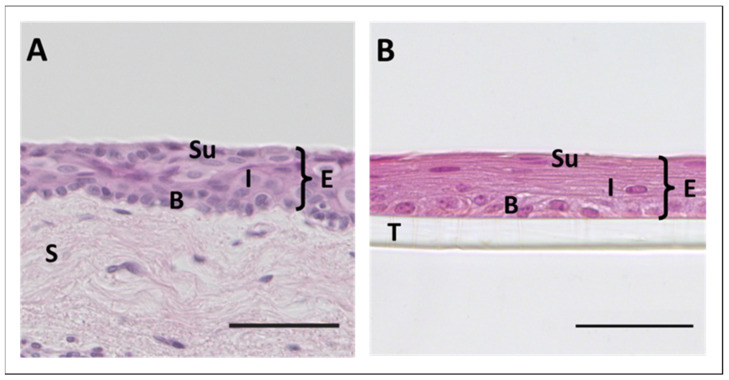
Development of 3D conjunctival epithelial cell culture model. (**A**) Hematoxylin and Eosin staining (HE) of human bulbar conjunctiva. (**B**) 3D cultured human conjunctival epithelial cells growing on a transwell insert. Isolation as explant culture on transwell with cultivation (21 days) and differentiation performed according to reference [89]. E = epithelium, Su = superficial epithelial cells, I = intermediate cells, B = basal cells, S = Stroma, T = transwell; Magnification is 200× (**A**) and 400× (**B**) with 50 µm scale bar.

As the conjunctiva covers most of the ocular surface area, conjunctival disorders can severely affect the quality of life [86,87,88]. Therapies based on reconstruction of the conjunctiva using 3D cell culture techniques, with conjunctival cells expanded in vitro on suitable matrices, are gaining increasing attention. However, the efficient culture of conjunctival cells and, in particular, differentiation and/or preservation of mature conjunctival cells and goblet cells pose special requirements for cultivation. Special attention should be paid to the choice of starting material when 3D cell cultures are established, as the three conjunctival regions show anatomical differences in terms of epithelial cell shape, stratification, and goblet cell density (see Section 2.2). In order to represent the physiological morphology in higher concordance, cell culture models should be established in 3D. To verify an ‘organ-like’ phenotype of cultured conjunctival cells, stratification (Figure 3) as well as cytokeratin 4, 13, and 19 expression are established as markers for mature conjunctival epithelial cells, while MUC5AC is the most common marker for identifying conjunctival goblet cells. A summary of current 3D conjunctiva and nasal cell culture models is presented in Table 1.

To study conjunctival cells, various species are used as donors including rabbits, bovids, and humans [89,90,91,92]. In addition, described cell culture conditions vary greatly with regard to isolation, medium, carrier matrices, and differentiation protocols. In most cases, isolation was carried out by enzymatic homogenization of the conjunctiva with subsequent seeding [90,91,92,93], but also attempts were made to use whole conjunctival pieces and isolate the cells by simply letting the explant culture grow out on a suitable matrix [89,94]. The majority of studies use medium based on Dulbecco’s Modified Eagle Medium (DMEM)/F12 nutrient mix with fetal bovine serum, insulin, and epidermal growth factor (EGF) as additives among others for the expansion of conjunctival cells (see Table A1, Appendix A). For differentiation into mature conjunctival epithelial cells and into goblet cells, special medium compositions as well as methods of air-liquid interface were evaluated, or both were applied in combination. 

Another approach to study conjunctival cell biology and effects of various therapies, but not as therapies per se, is the organ culture of conjunctiva pieces. In one study, it was shown that human-derived conjunctiva pieces could be cultivated long-term on gelatin sponges [95]. After 14 days of culture, a new conjunctival epithelium was formed with the expression of cytokeratin 19 and the presence of goblet cells. Of course, the mechanical stress on the tissue exerted under physiological conditions (blinking) should also be considered here and, ideally, the culture should be cultivated under mechanical stress, e.g., by means of a cell stretcher [95].

**Table 1:** 3D cell culture conditions of conjunctival epithelium.

#	Tissue Source	Goblet Cells	Cell Culture Conditions	Ref.
1	Rabbit-derived conjunctiva	yes	New Zealand white rabbit-derived palpebral and bulbar conjunctiva Isolation of conjunctival progenitor cells by homogenization of cells using collagenase and Trypsin-EDTA Expansion of conjunctival cells at 1 × 10^7^ cells/mL in bioprinted micro-constructs in conjunctival stem cells (CjSC)-medium Goblet cell differentiation was initiated by a special goblet cell differentiation medium for seven days No efforts were made to analyze the presence of mature conjunctival epithelium Goblet cells were detected by positive MUC5AC	[90]
2	Human-derived conjunctiva		Human donor-derived bulbar conjunctiva after cryopreservation for up to six months	[89]
		no	Isolation and expansion of conjunctival progenitor cells on feeder cells in conjunctival epithelial cells (CEC) medium by explant culture Expansion and differentiation by seeding 4.4 × 10^5^ cell/cm^2^ onto a decellularized porcine conjunctiva in a cell crown, submerged cultivation in CEC medium for 7 days followed by cultivation at air-liquid interface in differentiation medium Mature conjunctival cells were detected by stratification, morphology, and positive cytokeratin 19 staining No goblet cells were detected by MUC5AC staining	
		few	Isolation on decellularized porcine conjunctiva Isolation and expansion of conjunctival cells by direct outgrowth from an explant piece onto a scaffold Expansion on decellularized porcine conjunctiva placed in a cell crown, submerged cultivation in CEC medium for 7 days followed by cultivation at air-liquid interface in differentiation medium Mature conjunctival cells were detected by stratification, morphology, and positive cytokeratin 19 expression Goblet cells were detected by positive MUC5AC staining	
3	Bovine-derived conjunctiva	no	Bovine-derived bulbar conjunctiva Isolation of conjunctival epithelial cells by homogenization of cells using dispase and Trypsin-EDTA Expansion of conjunctival cells at 5 × 10^5^ cells/cm^2^ on collagen-coated transwell inserts in complete growth medium For 3D culture, cells were maintained at the air-liquid interface from day 5 onwards Mature conjunctival cells were detected by stratification, morphology, and cytokeratin 4 and 13 expression Goblet cells were not detected	[91]
4	Rabbit-derived conjunctiva	yes	New Zealand white rabbit-derived palpebral and fornical conjunctiva Isolation of conjunctival progenitor cells by homogenization of cells using dispase II and Trypsin-EDTA Expansion of conjunctival cells onto a collagen-coated cell culture dish in growth medium to sub-confluence For 3D culture, conjunctival cells were seeded at 1.1 × 10^6^ cells/cm^2^ in a temperature-responsive culture dish, and after 4 days, a second cell layer was seeded at 1.1 × 10^6^ cells/cm^2^ Mature conjunctival cells were detected by stratification, morphology, and cytokeratin 4 and 19 expression Goblet cells were detected by positive MUC5AC staining	[92]
5	Human-derived conjunctiva	yes	Human donor-derived biopsy specimens from superior temporal bulbar conjunctiva Isolation of conjunctival cells by homogenization using protease and differential attachment to plastic cell culture dishes Expansion of cells at 1.4 × 10^3^ cells/cm^2^ on plastic culture dishes in bronchial epithelial growth medium (BEGM) For 3D culture, cells were seeded at 2.1 × 10^4^ cells/cm^2^ onto transwell inserts in a mixed medium (BEGM/DMEM) and maintained at air-liquid interface from day 5 onwards Mature conjunctival cells were detected by stratification, morphology, and positive cytokeratin 19 staining Goblet cells were detected by positive MUC5AC staining	[96]
6	Human-derived conjunctiva		Human donor-derived conjunctiva from 6 different regions in initial experiments; the inferior fornical conjunctiva, as the best source of conjunctival stem cells, was then used in further 3D cell culture experiments Isolation of conjunctival cells by homogenization using Trypsin-EDTA	[93]
		no	Expansion on amniotic membrane at 1.7 × 10^4^ cells/cm^2^ with/without feeder cells in control medium (K) or without feeder cells in XerumFree (XF) or keratinocyte (SFM) medium for up to 7 days Mature conjunctival cells were detected by stratification, morphology, and positive cytokeratin 13 and 19 staining when cultured in K or XF; cells in SFM failed to attach to the amniotic membrane Goblet cells were not detected	
		no-few	Expansion on fibrin glue gel at 1.7 × 10^4^ cells/cm^2^ with feeder cells in control medium (K) or without feeder cells in XerumFree medium (XF) for up to 7 days Mature conjunctival cells were detected by stratification, morphology, and positive cytokeratin 13 and 19 staining when cultured in K or XF Goblet cells were not detected by MUC5AC staining, but few MUCS5AC transcripts were detected by qPCR	
7	Human-derived conjunctiva		Human donor-derived bulbar conjunctiva Isolation of conjunctival cells (epithelial cells and fibroblasts) by explant culture on plastic dishes in epithelial cell culture medium Preparation of a fibrin scaffold with 1 × 10^5^ fibroblasts/mL incorporated into the scaffold and 1 × 10^5^ epithelial cells/cm^2^ seeded onto the scaffold surface 24h after polymerization	[94]
		Few-no	Cultures of the scaffolds submerged in epithelial cell culture medium Mature conjunctival cells were detected by stratification, morphology, and positive cytokeratin 19 staining Goblet cells were detected by lectin staining of glycoconjugates (HPA) and MUC5AC ELISA at day 7, but no goblet cells were detected at day 14 by lectin staining of glycoconjugates (HPA)	
			Cultures of the scaffolds were maintained at the air-liquid interface from day 3 onwards in epithelial cell culture medium Mature conjunctival cells were detected by stratification, morphology, and positive cytokeratin 19 staining Goblet cells were detected by lectin staining of glycoconjugates (HPA) and MUC5AC ELISA at day 7	

DMEM: Dulbecco’s modified eagle medium, EDTA: ethylenediaminetetraacetic acid, ELISA: enzyme-linked immunosorbent assay, HPA: Helix pomatia agglutinin, MUC5AC: mucin 5AC, qPCR: quantitative real time polymerase chain reaction.

Different 3D cell cultures with nasal epithelial cells were previously described in the literature. Recent works by our group providing detailed information on the corresponding cell culture models (submerged, spheroid, organotypic 3D cultures, etc.) with the corresponding clinical applications were published previously [57,58,59]. Among others, the advantages of nasal epithelial cells over bronchial cells are discussed there. Because of their ease of collection (in contrast to bronchial cells, which are obtained by complex bronchoscopies), scientists can readily obtain nasal samples from patients and healthy donors and apply complex cell culture methods (reviewed in [58]). In addition, cell culture media, either commercially available or self-made, allow practical implementation of such cell cultures ex vivo (reviewed in [58]). Studies using nasal epithelial samples from patients with cystic fibrosis (CF) ensure important molecular insights [97]. Keegan and Brewington recently wrote about using these insights to guide individualized therapy for CF patients in their review [97]. The authors discuss that preclinical testing of drugs in such nasal epithelial cell cultures of CF patients should be performed to provide appropriate personalized therapy with Cystic Fibrosis Transmembrane Conductance Regulator (CFTR) modulators, because not every CF patient shows the same response to these therapeutics [97]. The establishment of nasal epithelial organoids may allow the implementation from simple dose-effect approaches to complex vector use with the major aim to help individuals who are suffering from serious diseases e.g., CF, nasal tumors. 

To the best of our knowledge, no study has reported the isolation, cultivation, or investigation of nasolacrimal epithelial cells. This could possibly be due to the limited accessibility of the nasolacrimal duct within its bony canal (nasolacrimal canal). Based on the similarities between the epithelial structures, we hypothesize that the cell culture technologies may be similar. However, this needs to be verified in future experiments.

### 3.3. Optical Coherence Tomography as a Useful Non-Invasive Technique for Ocular Surface Imaging and Disease Exploring

For several years, the increasing use of non-invasive methods to visualize structural depths and three-dimensional structures of the eye have been growing. In particular, OCT takes on an important role in ophthalmology (reviewed in [98]). 

In detail, the OCT is a non-invasive, non-contact, high-resolution optical methodology that can generate real-time cross-sectional images [98,99,100,101,102]. Currently, increasing adaptations and modifications of OCT are taking place, in which fast and better-resolution images can be generated [100]. These changes and technological achievements are due to a certain transition for several years between the traditional time-domain (TD) to Fourier-domain OCT (FD-OCT) methodology [100].

The FD-OCT technique uses the spectrum of reflected light to generate an image, which displays structural information on the ocular surface, cornea, and retina [100]. In contrast, previous OCT techniques used time-domain detection, an interferometer, and a scanning arm as well as a light-source of a low-coherence nature (reviewed in [103]). Napoli and colleagues summarized the usability of this instrument in their recent review [100], mapping the transition from the traditional to FD-OCT method. In addition, the same research group also tested the clinical applicability of OCT and highlighted its use in different areas, including in forensic medicine, where the authors studied postmortem ocular changes [98,101,104,105]. In another article, the same research group administered sodium carboxymethylcellulose over the cornea in healthy and dry eye patients and successfully measured adhesiveness using FD-OCT [106]. Moreover, Messner and colleagues demonstrated dry eye disease in a mouse model using OCT and fluorescein staining [107]. They concluded that fluorescein staining can detect defects of the corneal surface, but OCT can also provide information on the depth of the defect and the close layers [107].

In summary, OCT can provide important clinical information and was shown to be useful to date. As was also shown in appropriate animal models, the device is useful in basic research [72,73,108,109,110]. Such new technological achievements will certainly complement the complex culture experiments in the future. Thus, there should be a constant exchange of information with basic researchers and ophthalmologists who may develop further establishments and adaptations of OCT.

## 4. Clinically Relevant Molecular Applications of Conjunctival, Nasolacrimal, and Nasal 3D Cell Culture Models

In the previous section, we described the current state-of-the-art 3D cell culture technology for corneal, conjunctival, nasolacrimal, and nasal epithelial cells. These technologies can be used to perform functional studies that provide insight into common and distinct pathologies in these complex structures. Therefore, in this section we will exemplify three clinically relevant pathologies, namely measles virus infection, adenovirus infection, and allergies, which can affect both the eye and the nose.

### 4.1. Pathogenesis of Measles Virus Infection in the Eye and the Nasolacrimal Tract

**Figure 4:**
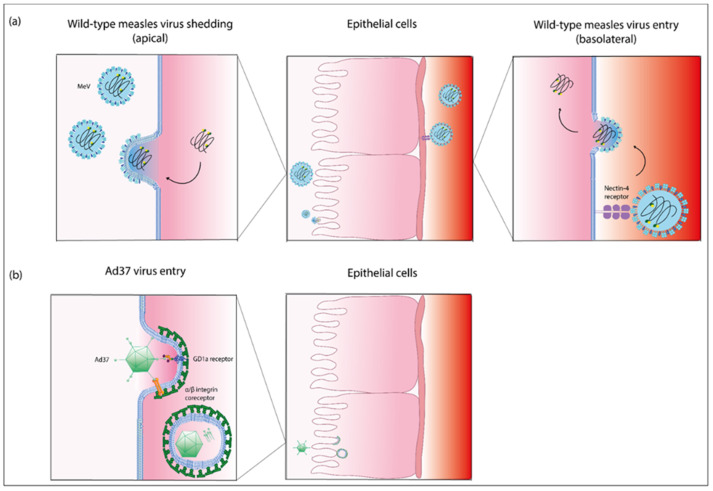
Entry and exit pathway of measles virus and entry pathway of adenovirus type 37. (**a**) Schematic representation of the entry and exit process of wild-type measles virus (MeV) in epithelia. MeV is transmitted to epithelial cells via the receptor nectin-4 located at the basolateral membrane. Entry occurs via membrane fusion. Progeny MeV particles bud from the apical surface of epithelial cells into the lumen [152,153]. This panel (**a**) was adapted from [111]. (**b**) Schematic representation of the entry pathway of adenovirus 37 (Ad37). Ad37 enters epithelial cells by binding the ganglioside (GD1a) receptor and α/β integrins as co-receptors. Cell entry occurs by clathrin-mediated endocytosis [141,154]. This panel (**b**) was adapted from [135]. Abbreviations: Ad, adenovirus; MeV, wild-type, i.e., pathogenic measles virus; GD1a, ganglioside. This figure was created with Adobe Inc. (2021) Adobe illustrator. Retrieved from https://adobe.com/products/illustrator, Dublin, Ireland, software purchased on 6 April 2021, link accessed last 6 April 2021, software last accessed on 12 October 2021).

Measles virus (MeV) is the causative agent of measles, one of the most transmissible human diseases. MeV is a negative-strand RNA virus of the *Paramyxoviridae* family [111]. The MeV genome has a size of approximately 16 kb; the size of the pleiomorphic virions ranges from 100 to 300 nm [112,113]. The entry receptors for pathogenic wild-type MeV are CD150/SLAM, expressed on immune cells, and epithelial nectin-4/PVRL4 [114]. After receptor binding, MeV fuses at the plasma membrane, and its exclusively cytoplasmic replication cycle is initiated (Figure 4a). MeV is transmitted through the respiratory route, and immune cells in the upper respiratory tract represent the primary target cells. MeV infection then spreads via immune cells within the lymphoid tissue, followed by viremia and systemic spread of the virus. In epithelia, infection occurs at the basolateral membrane; the virus then spreads from cell to cell and finally buds at the apical membrane [111,115,116]. Several studies on measles pathogenesis employ the macaque infection model [117], but human epithelial cell cultures have also been used [115,118]. Analyses of samples acquired during measles infection of children are also published [119,120]. These investigations yielded insights into the spread of MeV within the eye and nose. 

Conjunctivitis and coryza are hallmarks of measles [116] and shedding via the nasal mucosa is assumed to contribute to the high transmissibility of the virus [121]. Clinically, an ocular infection with MeV is thought to be acquired following viremia, leading to conjunctivitis and epithelial keratitis [122,123]. Measles infection is a major cause of childhood blindness in unvaccinated individuals, particularly in malnourished children [111]. During measles infection, MeV can be localized in ocular tissue [124] and its genomic RNA is detected in tear samples [125]. Detection of MeV antigen in epithelial cells within measles-associated conjunctival lesions was also reported previously [120,123]. In biopsies without conjunctival lesions, the antigen was only detected in the subepithelial tissue [120]. Syncytial giant cells as a characteristic feature of measles infection were also found in conjunctival swab samples during the early phase of the disease [119]. However, it is argued that these giant cells and ocular symptoms may represent sequelae of inflammation and the ensuing immune response [111,126]. 

Corneal rim epithelial cells can be infected with MeV after disruption of epithelial integrity [126] and eye protection as well as administration of measles convalescent serum into the conjunctival sac was reported to reduce the risk of measles infection [127,128,129]. This led some authors to the conclusion that the conjunctiva is a route of measles entry [127,128]. Nevertheless, ocular transmission is certainly not a main route of measles transmission, as opposed to transmission via respiratory droplets and aerosols [129].

In the macaque model of measles infection, high titers of cell-associated as well as cell-free virus were found in nasal swabs [130], and large groups of infected cells were observed in the nasal epithelium [131]. The release of infectious virus from macaque nasal epithelial cultures after both apical and basolateral inoculation has been reported [118]. These studies indicate that the nasal epithelium may play an important role in measles transmission. Syncytia were also observed in nasal secretions of children with measles [119,132]. A recent study using human airway epithelial cultures demonstrated transmission of infectious virus from epithelial to immune cells via dislodged epithelial infectious foci [133]. These findings support the notion of measles transmission via nasal secretions and sneezing. The pathogenesis of measles in the eye and nose is documented and studied in preclinical models and using clinical specimens. However, a distinct role for the communication between these organs in measles pathogenesis has not been established to date.

### 4.2. Pathogenesis of Adenovirus Infection in the Eye and the Nasolacrimal Tract

Human adenoviruses (HAdV) comprise more than 100 different types (http://hadvwg.gmu.edu/, accessed on 1 September 2021), which are divided into seven know species, HAdV-A to HAdV-G. As pathogens, adenoviruses cause a multitude of clinical diseases. Depending on the virus type, infections most frequently affect the respiratory and the gastrointestinal tract, as well as the eyes. An effective treatment is lacking, and the mortality rate in immune-compromised patients can amount to up to 70% [134]. HAdV infections in the eye manifest as either follicular conjunctivitis, pharyngoconjunctival fever (PCF), or epidemic keratoconjunctivitis (EKC) involving the cornea (reviewed in [135]). EKC represents one of the most frequent ocular diseases and is mainly caused by species D adenoviruses including HAdV-8, HAdV-37, HAdV-53, HAdV-54, HAdV-56, HAdV-64 (previously known as HAdV-19a), HAdV-82, and HAdV-85 [135]. Among these, HAdV-8, HAdV-19, and HAdV-37 cause the most severe conjunctivitis [136]. Pharyngoconjunctival fever (PCF) is mainly caused by HAdV-3 from species B, and HAdV-4 from species E is associated with PCF [137,138]. This infection mainly affects children, and outbreaks can occur in schools, kindergartens, and summer camps. Patients suffering from PCF usually present with mild symptoms such as follicular conjunctivitis, rhinitis, pharyngitis, and fever.

There is a limited number of studies analyzing HAdV infection in the nasolacrimal ducts and nasal epithelial cells. One study analyzed the susceptibility of primary human nasal epithelial cells to a broad range of different HAdV [139] and found that predominantly HAdV-5 derived from species C yielded robust transduction of these primary cells. Other non-species C viruses showed low transduction rates [139,140].

To study the pathogenicity of HAdVs associated with infections in the eye and efferent tear ducts, virus entry can be analyzed in respective infection models of the 3D cell culture model. Virus tropism is determined by primary receptors, and there is evidence in the literature that EKC-associated HAdV utilizes GD1a glycan [141] and CD46 [142] as an entry receptor. Furthermore, it was shown that various EKC-associated HAdVs utilize sialic acid containing glycans as cellular receptors [143]. In contrast, PCF-associated HAdVs enter cells utilizing the desmoglein 2 receptor (HAdV-3) [144] and the coxsackie- and adenovirus receptor (CAR, HAdV-4) [145]. Transduction of nasal epithelial cells was most efficient with HAdV-5 [139,140], which enters the target cell utilizing the CAR receptor [146]. After binding of the adenovirus fiber knob protein protruding from the adenovirus capsid to the primary receptor, the arginine-glycine-aspartic acid (RGD) motif of the adenovirus penton protein interacts with integrins of the host cell. It was shown that, for instance, HAdV-37 binds to α_V_β_1_ and α_3_β_1_ integrins for infection of corneal cells [147], which is followed by clathrin-mediated endocytosis (Figure 4b).

To study the pathogenesis of HAdV at the ocular surface or the nasolacrimal tract further, host species infection models in which infectious virus progeny can be observed were explored. Utilizing porcine corneal cell culture [148] and a three-dimensional culture model based on human corneal stromal fibroblasts [149], infections in the eye were analyzed. Moreover, alternative infection models for the eye immortalized human corneal epithelial cells were studied [150,151]. Furthermore, HAdV infection was explored in submerged 2D and organotypic 3D primary human nasal epithelial cells [59]. However, to date, studies investigating conjunctival epithelial and efferent tear duct epithelial susceptibility are missing.

### 4.3. Allergen Exposure in Ocular and Nasolacrimal Systems

The ocular and nasal epithelia may be affected in the setting of allergies [155,156]. This is a clinical example of how both compartments can be affected locally and how they are interconnected via allergen exposure (reviewed in [157]) [155,156]. Patients suffering from ocular allergic symptoms commonly have rhinitis symptoms, and both can be linked with asthma as well (reviewed in [158,159]. An effective medical treatment would reduce both ocular and nasal local symptoms simultaneously (reviewed in [160]) [155]. External triggers such as allergens can lead to rhino-conjunctival symptoms. About a quarter of the world’s population suffers from allergic diseases including hay fever, asthma, or atopic dermatitis [161], and circa 10–20% have an allergic rhinitis [162]. Epidemiologically, the highest disease rates are noticed in developed, westernized countries [163]. 

Rhinitis is characterized by runny nose/rhinorrhea, nasal congestion, itching, and/or sneezing due to underlying inflammation of the nasal mucosa [161,163,164,165]. Patients with an allergic rhinitis usually have conjunctivitis and asthma [161,166]. Allergic rhinitis is caused by an IgE-mediated reaction to inhaled substances [161]. Different guidelines are used for the classification of allergic/non-allergic rhinitis (for allergic rhinitis, the Allergic Rhinitis and its Impact on Asthma (ARIA) guidelines are commonly used) [161,163,167,168]. Nevertheless, there are still no molecular parameters which have been translated into clinical practice that can predict the type and severity of allergic rhinitis [161]. Allergies/atopy (allergic inflammation) can lead to a chronic rhinosinusitis (reviewed in [169,170]). Furthermore, allergens can also affect the eyes, which can be also a manifestation of allergic sensitization and can also appear together with rhinitis (=Allergic rhinoconjunctivitis) [171]. The ocular allergy affects the eyelid, the conjunctiva, and the cornea [172] and is observed either seasonally or as persistent symptoms throughout the year [172,173]. Due to the stimulation of the ocular and nasal mucosa by distinct allergens (e.g., outdoor allergens), clinically, increased tearing, redness, itching and swelling, runny or stuffy nose, as well as increased sneezing are characteristically observed [172,173,174].

**Figure 5:**
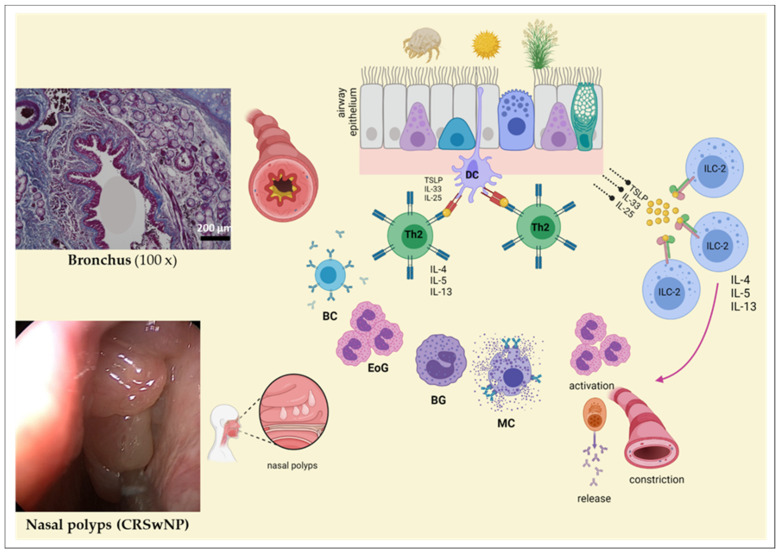
The influence of distinct triggers on the airway epithelium. Several triggers can affect or harm the epithelium, which consequently lead to a complex signaling cascade including the activation of T helper 2 (Th2) cells and the release of Th2 cytokines. Following cytokine secretion, several types of immune cells e.g., B cells (BC), eosinophil granulocytes (EoG), basophil granulocytes (BG), or mast cells (MC) are activated. In addition, through the direct stimulation of innate lymphoid cells type 2 (ILC-2), interleukin (IL)-4, IL-5, and/or IL-13 are also released. Clinically, through the activation of these cellular actors, mucus production, airway inflammation, and bronchoconstriction are observed in the bronchi. In addition, chronic inflammation in the nasal cavity leads to the development of nasal polyps. This figure was adapted from [175,176,186,187,188] and was created with BioRender.com. (left) Nasal polyps of a patient with a chronic rhinosinusitis and nasal polyps (CRSwNP). In this figure, polyps of the nasal mucosa are depicted from a patient with a chronic rhinosinusitis and allergy, who suffered from recurrent rhinitis and impediment of nasal breathing.

The underlying molecular mechanism is a T helper 2 (Th2)-polarized immune response, which is characterized by the secretion of cytokines such as interleukin (IL)-4, -5, or -13 (reviewed in [175,176]). When an allergen hits the epithelium, pro-inflammatory cytokines/alarmins such as IL-33, IL-25, or thymic stromal lymphopoeitin (TSLP) (reviewed in [175,176]) are secreted. On the one hand, these proteins can prime naïve T helper cells through dendritic cells via the MHC-II complex, which, as a result, then differentiate into mature Th2 effector cells (reviewed in [175,176]). On the other hand, these cytokines can also directly stimulate Innate Lymphoid Cells Type 2 (ILC-2), which are characterized by the release of type 2 cytokines (reviewed in [161,177,178,179]). These cytokines, in turn, then activate other immune cells, including, for example, B cells, basophil and/or eosinophil granulocytes, and mast cells (reviewed in [175,180]). Other lymphocytes such as regulatory T cells [177,180,181] and Th17 cells [182,183] also play an important role in this signaling cascade. Clinically, patients suffer from distinct local and systemic reactions due to an increased mucus production and inflammation (Figure 5).

A persistent Th2 inflammation can lead to the development of chronic sinusitis with nasal polyps (reviewed in [184]) (Figure 5). Due to the increased prevalence rate of patients with allergic rhinitis [185], which also influences quality of life [53], there must be more attention drawn to the search of biomarkers. Thus, it is necessarily important to investigate the development of new and complex cell culture technologies to increase the impact of the experimental performance and to implement disease-related hypotheses efficiently in vitro culture models.

## 5. Conclusions and Experimental Outlook

As evident from what is discussed above, the detailed morphological knowledge of the ocular and nasal epithelia stands in stark contrast to the lack of functional studies explaining physiological states and pathological alterations in these tissues and their interconnections. As one example, how infections (whether bacterial or viral) do not readily ‘jump over’ from the eye to the draining lacrimal ducts and from there to the nasal mucosa or take the reverse route, but usually remain localized, is incompletely understood. One reason could be the different morphology of the epithelial cells with their differential expression of receptors and distinct properties, which preclude pathogen spread across the regional epithelial borders. With the advent of modern technology and the invention of important methodological elements that are also used in the therapy of diseases, mammalian cell cultures represent essential tools to perform first pilot as well as complex experiments in vitro. Due to the high incidence of diseases associated with ocular and nasal epithelia, e.g., atopic disorders [189,190], advanced cell culture models representing these structures are key to advancing related approaches in molecular medicine. Starting from so-called submerged cultures, followed by 3D models to organ-like formats, different culture models are currently described in the literature. Technical improvements and refinements can make the testing of highly complex mechanistic hypotheses feasible ex vivo. This includes the investigation of the pathomolecular role of allergens (such as house dust mites or so-called ‘outdoor allergens’), but also the effects of viruses on corneal, conjunctival, efferent tear duct, and nasal epithelial cells. Exemplarily, AdV and MeV are important, as they can lead from harmless local symptoms to serious systemic diseases (reviewed in [111,191]). With the use of these cell culture technologies, entry receptors for AdV and MeV can be studied at the molecular level. In addition, AdV can be used in a next step as a potential gene therapy tool in these cell cultures with the intention to treat diseases curatively (reviewed in [192]). These viruses may also be used to develop novel anti-cancer agents for ocular and nasal malignancies with high medical need, since both MeV and AdV exhibit oncolytic properties [192,193,194,195,196]. Interestingly, the nose has already been positively evaluated to function as a potential vaccination site. Immunization via the nasal route is drawing increased interest, and the nasal cultures introduced in this review may provide a useful tool to study such vaccines [197,198,199]. Nasal and ocular cells can be used to perform co-cultures and thus interaction studies between cellular compartments, which can provide an additional layer of complexity, e.g., by enabling immunological observations. Practically, those co-cultures can be set up either with both nasal and ocular cells or co-cultures with immune cells, e.g., T lymphocytes. Moreover, also epigenetic and methylation patterns of (stimulated) nasal and ocular cells may provide key information for the characterization of potential molecular barcodes, from which mechanistic hypotheses regarding clinical phenomena can be established. First pilot experiments already demonstrate the functionality and robustness of these cultures with external stimulants including AdV or *Dermatophagoides pteronyssinus* (=house dust mite) [59,140]. Further establishment and more widespread use of the sophisticated techniques introduced herein to study the ocular and nasal epithelia will highlight their utility for medical progress for treatment and prevention of diseases with ocular and nasal pathologies.

## Data Availability

This review did not report any data.

## Appendix A

**Table A1:** Cell culture medium composition.

	Medium	Composition	Ref.
1	CjSC Medium	DMEM/F-12 (3:1) 10% fetal bovine serum 1% penicillin-streptomycin 1× insulin-transferrin-selenium 10 ng/mL epidermal growth factor 400 ng/mL hydrocortisone 0.1 nM cholera toxin 2 nM 3,3´,5-triiodo-L-thyronine 10 µM Y27632 1 µM A83-01 1 µM DMH1	[90]
	Goblet cell differentiation medium	Keratinocyte SFM 1% penicillin-streptomycin 50 µg/mL bovine pituitary extract 5 ng/mL epidermal growth factor 100 ng/mL bone morphogenetic protein 4 10 ng/mL fibroblast growth factor 10 100 ng/mL interleukin 13 1 µM A83-01	
2	CEC Medium	DMEM/F-12 (1:1) 10% fetal bovine serum 1% penicillin-streptomycin 5 µg/mL transferrin 10 ng/mL epidermal growth factor 400 ng/mL hydrocortisone 0.1 nM cholera toxin 0.075% sodium bicarbonate 0.18 mM adenin 5 µg/mL insulin 2 nM 3,3´,5-triiodo-L-thyronine	[89]
	Differentiation medium	DMEM/F-12 (1:1) 10% fetal bovine serum 1% penicillin-streptomycin 5 µg/mL transferrin 0.5 ng/mL epidermal growth factor 400 ng/mL hydrocortisone 0.1 nM cholera toxin 0.075% sodium bicarbonate 0.18 mM adenin 5 µg/mL insulin 2 nM 3,3´,5-triiodo-L-thyronine	
3	Complete growth medium	DMEM/F-12 (1:1) 10% fetal bovine serum 1% penicillin-streptomycin 2 mM L-glutamine 10 ng/mL epidermal growth factor 2 µg/mL hydrocortisone 0.1 µg/mL cholera toxin 50 µg/mL gentamycin 5 µg/mL insulin	[91]
4	Growth medium	DMEM/F-12 (1:1) 10% fetal bovine serum 1% penicillin-streptomycin 5 µg/mL insulin 5µg/mL transferrin 5 ng/mL selenium 10 ng/mL epidermal growth factor 10 ng/mL nerve growth factor	
5	Bronchial epithelial growth medium	Bronchial epithelial growth medium (BEGM) 5 µg/mL insulin 500 ng/mL hydrocortisone 500 ng/mL epinephrine 6.5 ng/mL triiodo-thyronine 10 ng/mL transferrin 10 ng/mL retinoic acid 0.13 mg/mL bovine pituitary extract 50 µg/mL:50 ng/mL gentamycin:amphotericin 10 ng/mL epidermal growth factor 0.15 mg/mL bovine serum albumin	[96]
	Mixture Medium	BEGM:DMEM (1:1) 5 µg/mL insulin 500 ng/mL hydrocortisone 500 ng/mL epinephrine 6.5 ng/mL triiodo-thyronine 10 ng/mL transferrin 10 ng/mL retinoic acid 0.13 mg/mL bovine pituitary extract 50 µg/mL:50 ng/mL gentamycin:amphotericin 0.5 ng/mL epidermal growth factor 0.15 mg/mL bovine serum albumin	
6	Control medium (K)	DMEM/F-12 (2:1) 10% fetal bovine serum 50 µg/mL penicillin-streptomycin 4 mM glutamine 5 µg/mL insulin 0.4 µg/mL hydrocortisone 0.18 mM adenine 8.1 µg/mL cholera toxin 2 mM triiodo-thyronine 10 ng/mL epidermal growth factor	[93]
	XerumFree medium (XF)	DMEM/F-12 (2:1) 10% XerumFree XF205 50 µg/mL penicillin-streptomycin 4 mM glutamine 5 µg/mL insulin 0.4 µg/mL hydrocortisone 0.18 mM adenine 8.1 µg/mL cholera toxin 2 mM triiodo-thyronine 10 ng/mL epidermal growth factor	
	Keratinocyte serum Free medium (SFM)	Keratinocyte SFM 50 µg/mL bovine pituitary extract 5 ng/mL epidermal growth factor	
7	Epithelial cell culture medium	DMEM/F-12 10% human serum 25 µg/mL fungizone 5000 U penicillin-streptomycin 1 µg/mL insulin 0.5 µg/mL hydrocortisone 2 ng/mL epidermal growth factor	[94]
